# Clinical manifestations of sporadic Creutzfeldt‐Jakob disease in public neurological center in Thailand

**DOI:** 10.1002/alz.088295

**Published:** 2025-01-03

**Authors:** Jedsada Khieukhajee, Narupat Suanprasert, Metha Apiwattanakul, Arada Rojana‐Udomsart, Tasanee Tantirittisak

**Affiliations:** ^1^ Neurological Institute of Thailand, Ratchathewi, Bangkok Thailand

## Abstract

**Background:**

Sporadic Creutzfeldt‐Jakob disease (sCJD) is one of common causes of rapidly progressive dementia worldwide. However, because of the variety of its clinical presentations that mimic other cognitive disorders, the certain diagnosis in public hospitals is still limited. Therefore, this study provides more information about disease manifestations and clinical courses of probable sCJD cases in Thailand.

**Method:**

The retrospective cross‐sectional study of probable sCJD patients visited Neurological Institute of Thailand during 2018‐2023 was conducted. Probable sCJD cases were diagnosed according to 2017 International CJD Surveillance Network Diagnostic Criteria. Demographic data, clinical presentations, brain MRI, EEG, CSF analyses, and clinical course were reviewed.

**Result:**

The total of 17 probable sCJD cases were studied. There was 11 female (64.71%) with the median age of 62 (IQR 14.5) years. The median onset of symptom was 2 months (IQR 2) before hospital visit, which cognitive impairment is the most common first presentation (29.41%) followed by ataxia (23.53%) and visual disturbances (17.65%). Myoclonus was found in most patients during the disease course at 2 weeks prior to diagnosis. Most patients had typical high signal intensities at both caudate/putamen and cortical regions (76.47%). Generalized periodic discharges with triphasic morphology of 1‐1.5 Hz were found in 11/14 patients with EEG results (78.57%). Most patients progressed to akinetic mutism and bed‐bound within 1 month after hospital visit.

**Conclusion:**

sCJD has diverse clinical presentations that resemble other cognitive disorders. Detailed assessment of signs and symptoms together with proper investigations could help to differentiate this condition in resource‐limit setting.

